# Practical Departments

**Published:** 1904-08-20

**Authors:** 


### PRACTICAL DEPARTMENTS.

HALL'S SANITARY WASHABLE DISTEMPER.

(Sissons Brothers and Co., Limited, Hull.)

We can thoroughly testify to the excellence of Hall's Sanitary Washable Distemper as a medium for painting walls, etc. The colours are good and cover a wide range, and the distemper is equally adaptable for interior or exterior decoration. A feature which renders the material indispensable in schools, hospitals, and similar institutions is the fact that the medium is not only washable, but is undamaged by the application of chemical disinfectants, which may be applied by means of a spray or by cloths soaked in the particular antiseptic selected. The periodic disinfection of the walls of many institutions carried out in this way is highly desirable, and at the same time is frequently neglected on account of the unsuitability of the wall-coverings, a difficulty which the use of Hall's distemper will avoid.

